# Description of a new species of *Wormaldia* from Sardinia and a new *Drusus* species from the Western Balkans (Trichoptera, Philopotamidae, Limnephilidae)

**DOI:** 10.3897/zookeys.496.9169

**Published:** 2015-04-16

**Authors:** Simon Vitecek, Ana Previšić, Mladen Kučinić, Miklós Bálint, Lujza Keresztes, Johann Waringer, Steffen U. Pauls, Hans Malicky, Wolfram Graf

**Affiliations:** 1 Department of Limnology and Oceanography, University of Vienna, Althanstrasse 14, A-1090 Vienna, Austria; 2 Department of Biology, Faculty of Science, University of Zagreb, Rooseveltov trg 6, HR-10000 Zagreb, Croatia; 3 Senckenberg Biodiversity and Climate Research Centre (BiK-F), Senckenberganlage 25, D-60325 Frankfurt a.M., Germany; 4 Hungarian Department of Biology and Ecology, Babeş-Bolyai University, Clinicilor 5–7, 400006 Cluj-Napoca, Romania; 5 Sonnengasse 13, Lunz am See, Austria; 6 Institute of Hydrobiology and Aquatic Ecology Management, University of Natural Resources and Life Sciences, Max Emanuel-Strasse 17, A-1180 Vienna, Austria

**Keywords:** Caddisfly, Europe, larval key, taxonomy, conservation, Mediterranean, hydropower

## Abstract

New species are described in the genera *Wormaldia* (Trichoptera, Philopotamidae) and *Drusus* (Trichoptera, Limnephilidae, Drusinae). Additionally, the larva of the new species *Drusus crenophylax* **sp. n.** is described, and a key provided to larval *Drusus* species of the *bosnicus*-group, in which the new species belongs. Observations on the threats to regional freshwater biodiversity and caddisfly endemism are discussed.

The new species *Wormaldia sarda* **sp. n.** is an endemic of the Tyrrhenian island of Sardinia and differs most conspicuously from its congeners in the shape of segment X, which is trilobate in lateral view. The new species *Drusus crenophylax* **sp. n.** is a micro-endemic of the Western Balkans, and increases the endemism rate of Balkan Drusinae to 79% of 39 species. Compared to other Western Balkan *Drusus*, males of the new species are morphologically most similar to *Drusus discophorus* Radovanovic and *Drusus vernonensis* Malicky, but differ in the shape of superior and intermediate appendages. The females of *Drusus crenophylax* **sp. n.** are most similar to those of *Drusus vernonensis*, but differ distinctly in the outline of segment X. Larvae of *Drusus crenophylax* sp. n. exhibit toothless mandibles, indicating a scraping grazing-feeding ecology.

## Introduction

The Mediterranean area is a flora and fauna biodiversity hot-spot. The Tyrrhenian islands and the Balkans, in particular, are noteworthy for their high number of plant endemics (Médail and Quezél 1997, 1999; Nikolić et al. 2008; Fenu et al. 2010; Bacchetta et al. 2012), and mammal and invertebrate endemics (Holdhaus 1924, Vigne 1992, Muccedda et al. 2002, Griffiths et al. 2004, Grill et al. 2007). Freshwater biodiversity has recently become a focus of attention throughout Europe, including the Mediterranean region with the Western Balkans and Sardinia (e.g., di Sabatino 2003, Zakšek et al. 2009, Tierno de Figueroa et al. 2013, Klobučar et al. 2013, Weiss et al. 2014).

The genus *Wormaldia* currently comprises 204 species (Morse 2014) of which 36 species occur in Europe (Malicky 2005, Graf et al. 2008). Most species are widely distributed, but also several apparently highly endemic species have been described (Graf et al. 2008, Martínez-Menéndez and González 2011). Aquatic stages of the genus, with few exceptions, prefer crenal and rhithral sections of alpine to lowland streams, are caseless and behave as passive filter feeders using characteristic nets (Graf et al. 2008). Species in the genus exhibit characteristic male genitalia, but also comparatively high variability, particularly of the phallic structures (Malicky 2004, Martínez-Menéndez and González 2011, Neu pers. comm.), resulting in the description of several subspecies.

The genus *Drusus* is in the subfamily Drusinae Banks, and comprises 84 species (Malicky 2004, 2005; Kučinić et al. 2011a; Oláh 2010, 2011; Oláh and Kovács 2013). Larvae of the group prefer eucrenal to epirhithral sections of cold alpine or montane streams and brooks. Feeding ecology of *Drusus* larvae is complex, and three different feeding guilds can be distinguished based on the shape of larval mandibles and leg setation: filtering carnivores, omnivorous shredders, and scraping grazers (Pauls et al. 2008, Graf et al. 2009). Taxonomic richness of Drusinae is particularly high in the Western Balkans, including a high number of micro-endemics (Malicky 2004; Graf et al. 2008; Oláh 2010, 2011; Kučinić et al. 2011a, b; Oláh and Kovács 2013, Previšić et al. 2014a, b).

In this paper we describe a new species of *Wormaldia* and a new grazer *Drusus* species, including a key to the hitherto known larval stages of the *bosnicus*-group, in which *Drusus crenophylax* sp. n. belongs.

## Materials and methods

Adults were collected using sweep nets and immature stages by handpicking. Collected specimens were stored in 70% and 96% EthOH, for morphological and molecular analyses, respectively.

Male and female genitalia were examined after being cleared in either KOH or lactic acid. Nomenclature of male genitalia of *Wormaldia* McLachlan follows Nielsen (1957, for *Wormaldia occipitalis* Pictet), nomenclature of male genitalia of *Drusus* follows Nielsen (1957, for *Limnephilus flavicornis* Fabricius) using the simplifying terms “superior appendages” for the lateral processes of segment X (cerci *sensu* Snodgrass 1935), and “intermediate appendages” for the sclerite and the anterior process of segment X (paraproct *sensu* Snodgrass 1935). Nomenclature of larval morphological features follows Wiggins (1998) and Waringer and Graf (2011), nomenclature of primary setae and setal areas follows Wiggins (1998). Illustrations were prepared according to Thomson and Holzenthal (2010) in which pencil drawings made with a camera lucida are digitized, edited and inked in Adobe Illustrator (v. 16.0.4, Adobe Systems Inc.).

Molecular genetic sequence data were used to support larval association and assess relationships to previously described *Drusus* species. DNA extraction and amplification of a 541-bp-long fragment of the mtCOI gene using standard primers (forward primer: Jerry, Simon et al. 1994, reverse primer: S20, Pauls et al. 2006) was performed as outlined by Pauls et al. (2008) and Previšić et al. (2009b). Sequences were edited manually using Geneious version R7 (http://www.geneious.com, Kearse et al. 2012) and aligned using MAFFT (Katoh and Standley 2013). Sequences were deposited in GenBank under Accession nos: KC881524, KP793081–KP793089 (Table 1). Inter- and intraspecific genetic distances (uncorrected *p*-distances) were calculated in Mega 4.0.1 (Tamura et al. 2007).

**Table 1.** Detailed list of *Drusus* specimens used for mtCOI analysis. Abbreviations: M adult male, F female; L larva; U unknown.

Species	Locality	Specimen ID/Stage	Accession #	Collectors
*Drusus crenophylax*	44°32.932'N, 17°23.562'E	fDsp4501M/M	KP793082	Dmitrović, Šukalo
*Drusus crenophylax*	44°33.003'N, 17°23.580'E	fDsp4502L/L	KP793083	Dmitrović, Šukalo
*Drusus crenophylax*	44°33.003'N, 17°23.580'E	fDsp4503L/L	KP793081	Dmitrović, Šukalo
*Drusus crenophylax*	44°33.003'N, 17°23.580'E	fDsp3401F/F	KP793084	Dmitrović, Šukalo
*Drusus crenophylax*	44°33.003'N, 17°23.580'E	fDsp3402F/F	KP793085	Dmitrović, Šukalo
*Drusus vernonensis*	41°0.887'N, 21°10.448'E	DdphPEIM1/M	KC881524	Kučinić, Graf
*Drusus vernonensis*	41°0.887'N, 21°10.448'E	DdphPEIM2/M	KP793087	Kučinić, Graf
*Drusus vernonensis*	41°0.887'N, 21°10.448'E	DdphPEIM3/M	KP793086	Kučinić, Graf
*Drusus discophorus*	Macedonia, Jablanica Mts.	fDds0110M/M	KP793089	Kučinić
*Drusus discophorus*	Macedonia, Jablanica Mts.	fDds0112F/F	KP793088	Kučinić

## Taxonomy

### Wormaldia sarda

Taxon classificationAnimaliaTrichopteraPhilopotamidae

Graf & Malicky sp. n.

http://zoobank.org/F02C5CF5-9043-463F-809B-FCD5D2B8FBD2

#### Material examined.

**Holotype.** 1 male pupa, holotype: Sardinia, Gola di Gorruppo; 40°11.122'N, 9°30.104'E; 350 m a.s.l.; 28.03.2001; leg. Monika Hess, Ulrich Heckes; currently in coll. W. Graf, will deposited in the Biologiezentrum des Oberösterreichischen Landesmuseums, Linz, Austria.

#### Type locality.

Italy, Sardinia.

#### Diagnosis.

Morphology of the male terminalia suggests placement of the new species in *Wormaldia*. The species is unique in the European Trichoptera fauna, and easily differentiated from all other *Wormaldia* species by the combination of the following characters: (1) presence of median subtriangular protrusion in the distal half of the harpago, (2) membraneous dorsoproximal portion and trilobate lateral portions of segment X, and (3) distinct sclerotized structures visible on the invaginated phallus.

#### Description.

*Adults* (in pupa). Habitus dark, sclerites and tergites brown; cephalic and thoracic setal areas pale; cephalic, thoracic and abdominal setation dark brown; legs light brown, proximally darker; haustellum and intersegmental integument pale cream. Wings brown mottled with golden patches. Male maxillary palp 5-segmented. Spurformula 2–4–4 in males.

**Figure 1.**
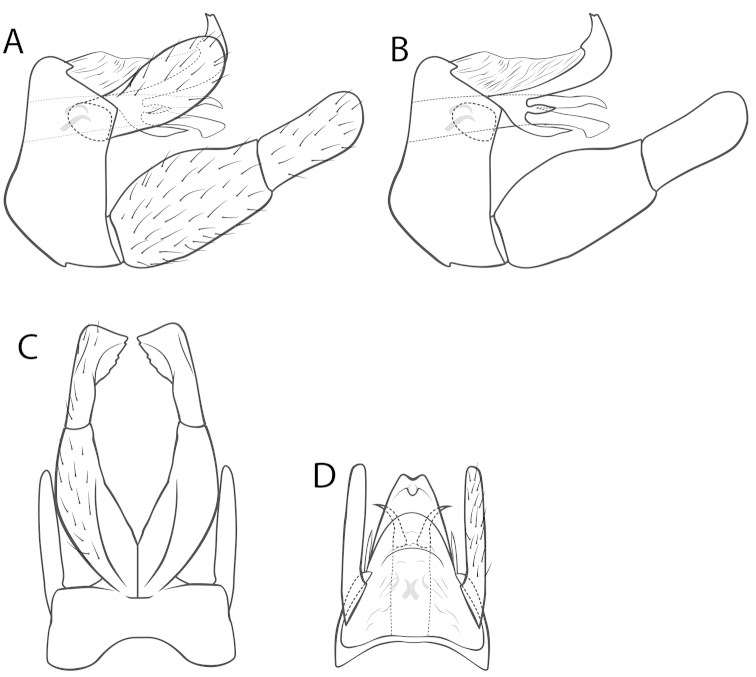
Male genitalia of *Wormaldia sarda* sp. n. **A** right lateral view, intact **B** right lateral view, superior appendage removed **C** ventral view **D** dorsal view.

*Male genitalia* (Fig. 1A–D). Segment IX in lateral view subrectangular, bulging anteriad; dorsal quarter reduced to a narrow transverse bridge, ventral 3/4ers broad (Fig. 1A). Segment X in lateral view trilobate: unpaired dorsal lobe strongly convex with a bicuspid apex, dorsoproximally membraneous; 1 lateromedian lobe, subovate, pointed on either side; 1 ventral lobe, posteriad, pointed on either side (Fig. 1A, B). Superior appendages suboval, curved dorsad in lateral view, flat with a rounded apex in dorsal and ventral view (Fig. 1A, C, D). Invaginated phallus terminally with a dorsal pair of sclerotized, laterad divergent tines and a ventral sclerotized plate; internally with 4 distinct tines (Fig. 1A, D). Coxopodite subovate in lateral view, ventrally with a sharp mediolaterad ridge (Fig. 1A, C). Harpago subovate in lateral view, in ventral view distally with a median subtriangular serrated protrusion flattened dorsoventrally (Fig. 1A, D).

**Figure 2.**
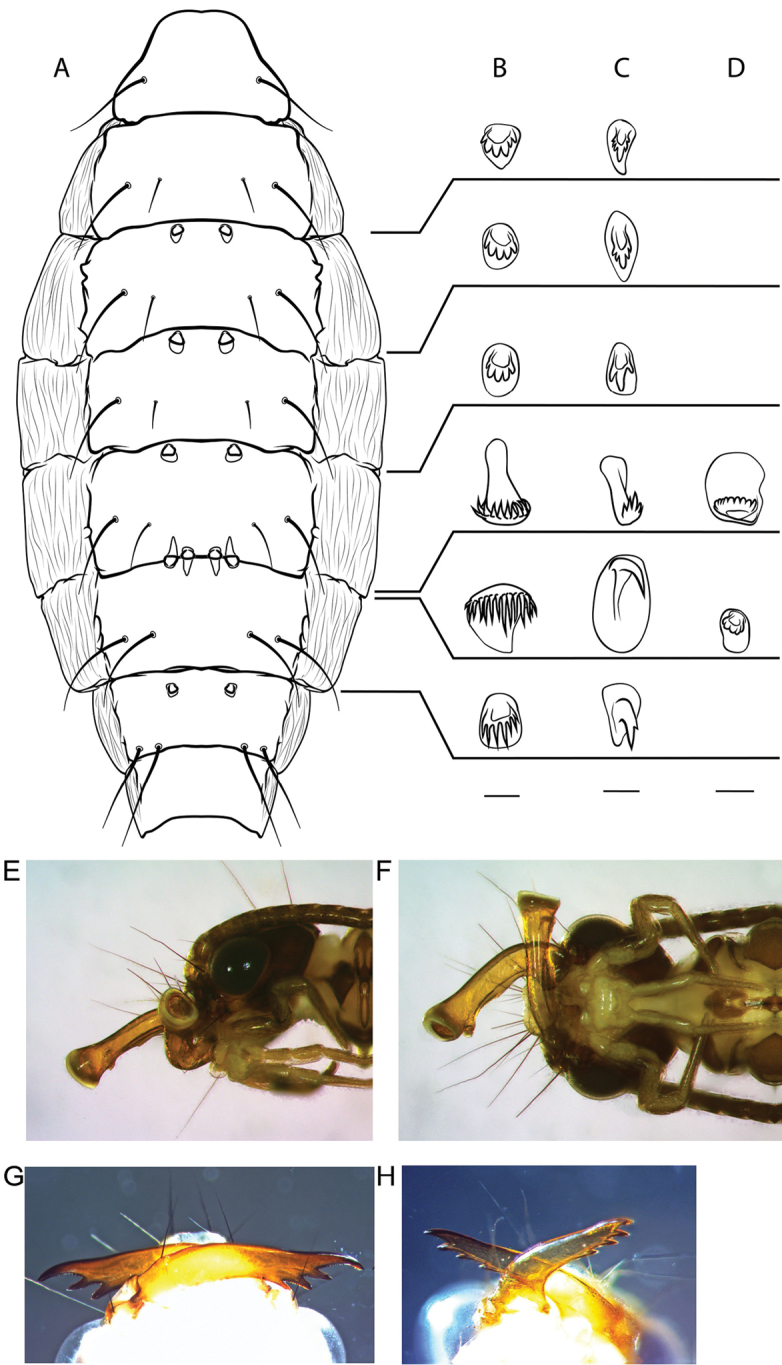
Pupal characteristics of *Wormaldia sarda* sp. n., *Philopotamus montanus*, and *Wormaldia* spp. **A** generalized pupal abdomen in dorsal view, depicting the position of the dorsal sclerites **B** dorsal sclerites of *Philopotamus montanus* **C** dorsal sclerites of *Wormaldia occipitalis* **D** dorsal sclerites of *Wormaldia sarda* sp. n. **E** head of *Wormaldia sarda* pupa in left lateral view **F** head of *Wormaldia sarda* pupa in ventral view **G** pupal mandibles of *Philopotamus montanus* in ventral view **H** pupal mandibles of *Wormaldia copiosa* in ventral view. Scale bars: 100 µm (**B**); 50 µm (**C, D**).

*Mature pupa* (Fig. 2D–F). Mandibles tubular, dilated at the apex (Fig. 2E,F). Abdominal dorsal sclerites as in Fig. 2D.

Female and larva unknown.

#### Etymology.

The species epithet refers to the island of Sardinia, the type locality.

### Drusus crenophylax

Taxon classificationAnimaliaTrichopteraLimnephilidae

Graf & Vitecek sp. n.

http://zoobank.org/4FBB2D55-59BD-46AB-8E39-B34F2D892C79

#### Material.

**Holotype.** 1 male: Bosnia and Herzegovina, Cvrcka river; 44°32.932'N 17°23.562'E; 393 m a.s.l.; 01.10.2014; leg. Dejan Dmitrović, Goran Šukalo; specimen identifier: fDsp4501M. Paratypes: 2 females: Bosnia and Herzegovina, Spring of Cvrcka river, Vilenjska vrela; 44°33.003'N, 17°23.580'E; 456 m a.s.l.; 12.09.2012; leg. Dejan Dmitrović; specimen identifiers: fDsp3401F, fDsp3402F. 4 males, 3 females, 19 larvae: Bosnia and Herzegovina, Spring of Cvrcka river, Vilenjska vrela; 44°33.003'N, 17°23.580'E 456 m a.s.l.; 12.09.2012; leg. Dejan Dmitrović, Goran Šukalo; specimen identifiers for 3 larvae: fDsp4502L, fDsp4503L, fDsp4504L. Holotype and paratypes currently in coll. W. Graf, will deposited in the Biologiezentrum des Oberösterreichischen Landesmuseums, Linz, Austria.

#### Type locality.

Bosnia and Herzegovina, Republika Srpska, Cvrcka River.

#### Diagnosis.

Males of the new species are most similar to *Drusus discophorus* Radovanovic and *Drusus vernonensis* Malicky, but exhibit (1) subtriangular superior appendages in lateral view, (2) subtriangular, low tip of the intermediate appendage in lateral view, and (3) simple, rounded tips of intermediate appendages in caudal view. *Drusus discophorus* males have suboval superior appendages and a high round tip of the intermediate appendage in lateral view; *Drusus vernonensis* males have round superior appendages in lateral view and trilobate tips of intermediate appendages in caudal view.

Females of the new species show the reduced median lobe of the vaginal sclerite and high base of the lateral lobe of segment IX as typical for Balkan Drusinae, and are most similar to *Drusus vernonensis*, but exhibit (1) a sharp dorsal notch of segment X in lateral view, and (2) segment X with 2 round median lobes in dorsal view. *Drusus vernonensis* females have a rounded dorsal outline of segment X and lack the median lobes of segment X.

Larvae of the new species are most similar to *Drusus klapaleki* Marinković-Gospodnetić and *Drusus serbicus* Marinković-Gospodnetić, but exhibit (1) a semicircular area dorsomedially on the pronotum anterior the pronotal ridge void of white recumbent setae, (2) lateral gills, and (3) a subtriangular pronotal ridge in lateral view. Larvae of *Drusus klapaleki* have white recumbent setae covering the whole pronotum, and larvae of *Drusus serbicus* lack lateral gills and have an annular pronotal ridge.

#### Description.

*Adults*. Habitus dark; sclerites and tergites brown; cephalic and thoracic setal areas pale; cephalic, thoracic and abdominal setation blond; legs light brown to fawn, proximally darker; haustellum and intersegmental integument pale, whitish. Wings smoky, with dark setae. Male maxillary palp 3-segmented. Forewing length 11–13.2 mm, spur formula 1–3–3 in males; forewing length 13–14.5 mm, spur formula 1–3–3 in females.

**Figure 3.**
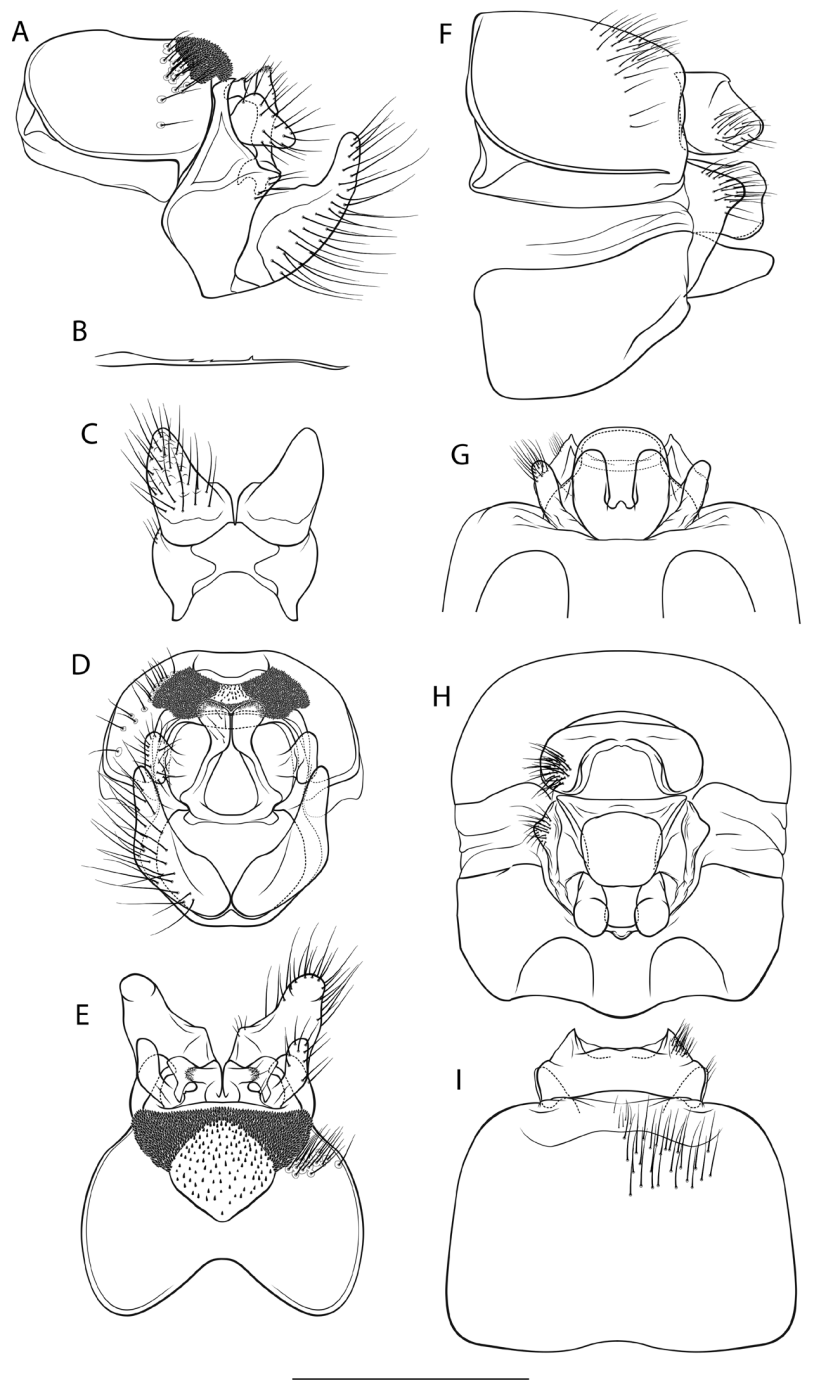
Genitalia of *Drusus crenophylax* sp. n. **A–E** male genitalia: **A** right lateral view **B** paramere in right lateral view **C** ventral view **D** caudal view **E** dorsal view **F–I** female genitalia: **F** right lateral view **G** ventral view **H** caudal view **I** dorsal view. Scale bar: 1 mm.

*Male genitalia* (Fig. 3A–E). Tergite VIII dark brown, in dorsal view cranially distinctly incised, with lighter areas around fused alveoli; setation concentrated at laterocranial borders of spinate areas; spinate area as two ± triangular laterocaudal lobes medially connected by a band of spines, embracing a medial, indent less sclerotized area (translucent in cleared specimens) with scarce spines. Ninth abdominal segment (IX) ventrally wider than dorsally in caudal view; in lateral view medially with a sharp caudad protrusion and a ventral protrusion, embracing the base of the inferior appendices. Superior appendages in lateral view subtriangular, somewhat Y-shaped with a shorter dorsal and a longer ventral protrusion separated by a slight indentation. Intermediate appendages in lateral view blocky with 2 tips, the proximal sharp, the distal high, rounded, rough; in dorsal view the tips parallel, extending laterally: a bar-shaped, laterally rounded distal tip and a sharp proximal tip, separated by a rounded excision with round edges; in caudal view approximately triangular, tips rounded. Inferior appendages (gonopods *sensu* Snodgrass 1935) in lateral view proximally wide, medially slightly constricted with a slight dorsal triangular protrusion, curved dorsadly in the slender posterior third; in dorsal, ventral and caudal view proximal part laterad, distal part approximately straight in dorsoventral plane, curved dorsad; in caudal view tips distinctly slender; setal alveoli fused, creating a rugged, less sclerotized ventral area. Parameres simple, with a distinct medial thorn-like spine and 2 proximal spines in the proximal half.

*Female genitalia* (Fig. 3F–I). Segment IX setation abundant, concentrated in the caudal half; lateral lobe of segment IX membraneous, in lateral view oblique triangular, the ventral edge about twice as long as the dorsal edge, with a dorsal sclerotized setose part protruding caudally; in dorsal and ventral view slender, projecting lateradly; in caudal view dorsal sclerotized setose part somewhat triangular. Segment X in lateral view with a proximal and a distal part, defined by a sharp dorsal notch; in dorsal view trapezoidal, with rounded shoulders, 2 small dorsal median lobes, and distally with 2 triangular, sharp-tipped lateral lobes, each with a lateral rounded setose and a small median rounded protrusion; ventrally unsclerotized, open. Supragenital plate in lateral view sinuously-edged quadrangular with a small, rounded dorsal protrusion, caudal line slightly indent; in ventral view quadrangular, in caudal view quadrangular, dorsally slightly wider than ventrally. Vulvar scale in lateral view triangular, rather straight, longer than the supragenital plate; in ventral view slender with 3 lobes: 2 lateral lobes, digitiform, roundly oval, straight; 1 median, short (reduced), of greater width than length: length approximately 1/6th of that of lateral lobes.

**Figure 4.**
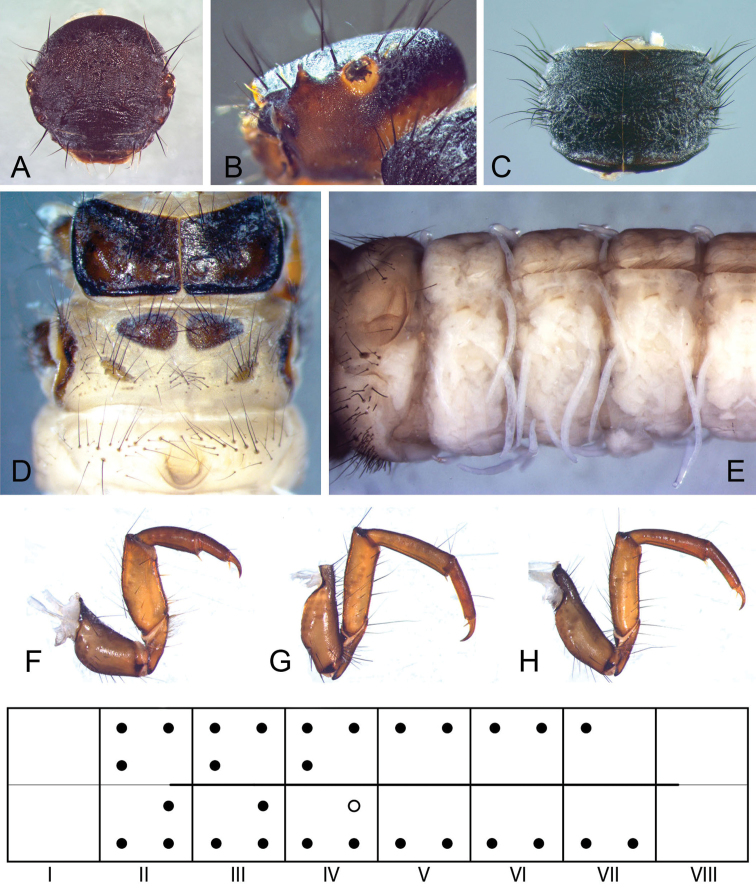
Larval characteristics of *Drusus crenophylax* sp. n. **A** head, frontal view **B** head, left lateral view **C** pronotum dorsal view **D** meso- and metathorax with abdominal segment I, dorsal view **E** abdominal segments I-V, left lateral view **F** left thoracic leg I, frontal view **G** left thoracic leg II; frontal view **H** left thoracic leg III, frontal view; bottom, gill and lateral line diagram, positions of gills are depicted as black circles, position of lateral line bold.

*Fifth instar larva* (Fig. 4A–I). Head capsule hypognathous, finely granulated with a field of microspinules dorsal to each eye, dark brown dorsally, fading to yellow ventrally; 18 pairs of primary setae present: #1, 4, 6, 10, 12, 13 yellow and #6, 13 short, inconspicuous, the rest dark brown, long (Fig. 4A); antennae located on high carinae, each carina about as high as long, both strongly curved mediad (Fig. 4B); mandibles toothless. Pronotum dark brown, coarsely granulated; distinct medial ridge present, rounded, steeper anteriorly in lateral view; recumbent white setae present, but lacking in a semicircular area anterior the pronotal ridge (Fig. 4C); pronotal horn present. Mesonotum completely covered by 2 sclerites, dark brown, with darker apodemes; edges black; *sa*1 comprising 4–6 setae, *sa*2 and *sa*3 connected, comprising 28–34 setae in total on each sclerite (Fig. 4D). Metanotum with 3 pairs of sclerites: anteriomedian sclerites subtriangularly ovoid, dark brown with 11–19 setae; posteromedian sclerites rhomboid, pale brown, with 13–15 setae; lateral sclerites long, curved dorsally in lateral view, pale brown fading to yellow ventrally with a dark median spot and 21–25 setae (Fig. 4E). Legs yellow-light brown, dorsally and distally darker (Fig. 4F–H). Abdomen white (Fig. 4G), dorsal gills from II praesegmental position to VI praesegmental position, lateral gills from II praesegmental position to IV praesegmental position, ventral gills from II prasegmental position to VII postsegmental position; lateral line from last quarter of II to first quarter of VIII (Fig. 4I); abdomen I with 1 dorsal and 2 lateral protuberances, posterior sclerites absent on lateral protuberances, setal areas *sa*1–3 fused dorsally and ventrally (Fig. 4D, E), sternum bearing 2 setae with distinct basal plates; abdomen VIII with 2 long and 2–4 short posterodorsal setae on either side; abdomen IX with 1 posterodorsal seta on either side, dorsal sclerite IX semicircular, pale brown with 7 long and several shorter setae. Case simple, constructed of mineral particles.

#### Molecular species delimitation and larval affiliation.

**Figure 5.**
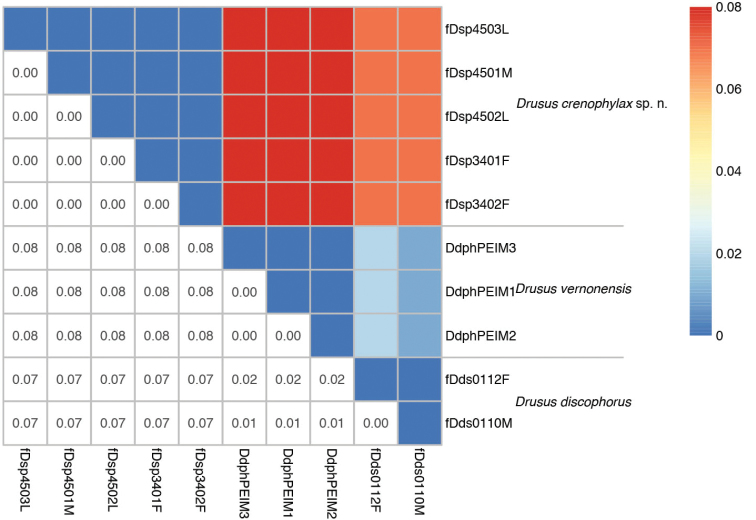
Distance matrix (lower left) and colour heat map (upper right) showing uncorrected inter- and intraspecific *p*-distances of the partial mtCOI sequence (541 bp) between *Drusus crenophylax* sp. n., *Drusus vernonensis* and *Drusus discophorus*. For detailed information on the haplotypes, see Table 1.

Analysis of the genetic distance of mtCOI between *Drusus crenophylax* sp. n. and the in the adult stage morphologically most similar species, *Drusus discophorus* and *Drusus vernonensis*, clearly supports the recognition of the new species. Uncorrected *p*-distances recorded in a fragment of the mtCOI gene (ranging from 2–8%; Fig. 5), agree with the interspecific distances commonly recorded in Limnephilidae (e.g., Graf et al. 2005; Kučinić et al. 2011a; Previšić et al. 2014a, b) and other caddisfly families (e.g., Hydropsychidae; Pauls et al. 2010). Also, all haplotypes of *Drusus crenophylax* sp. n. adults were completely identical to another and those of undescribed *Drusus*-larvae collected at the locus typicus, enabling confident affiliation of larvae and adults of *Drusus crenophylax* sp. n.

#### Ecology and distribution.

**Figure 6.**
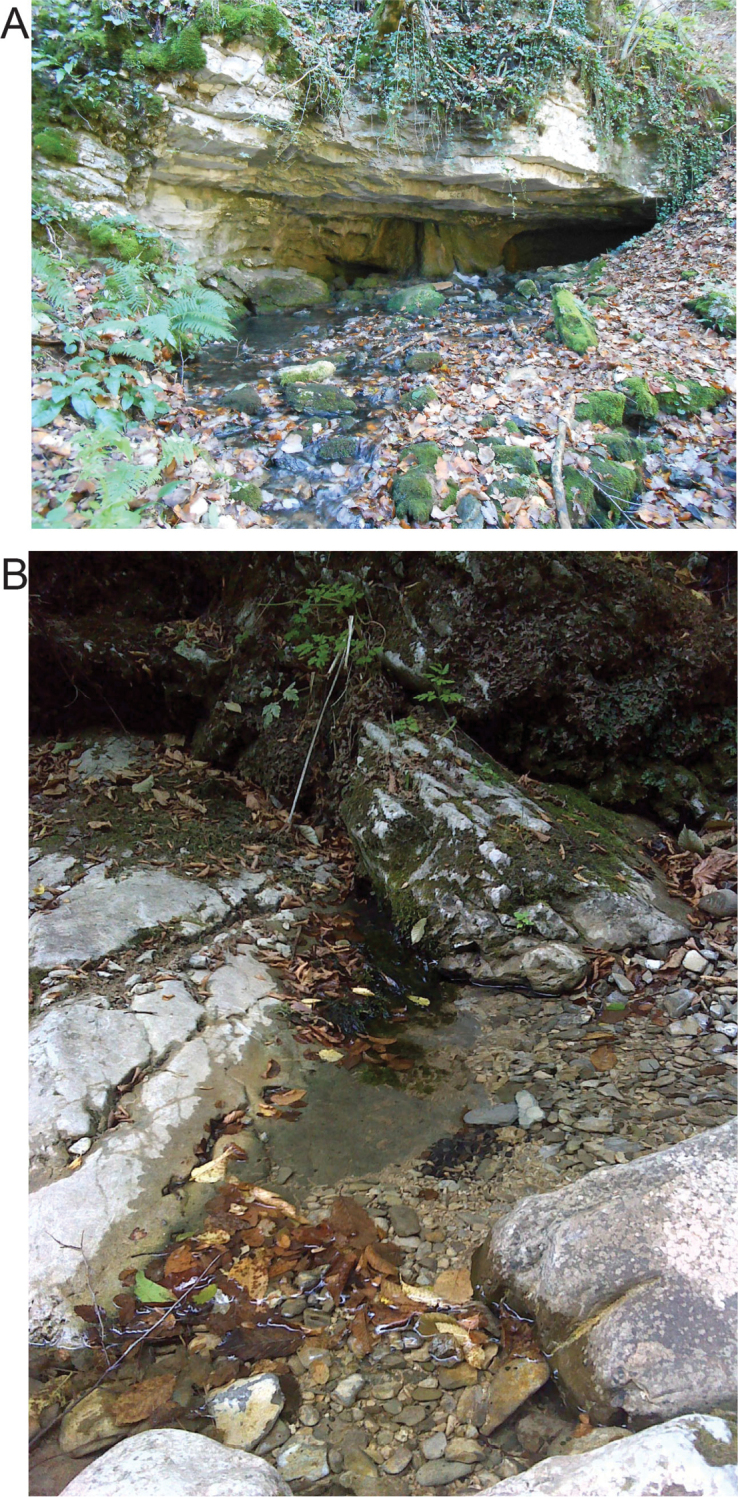
Habitat of *Drusus crenophylax* sp. n. at the type locality. **A** collection site of the larval paratypes **B** collection site of the male holotype.

Drusinae species typically are members of crenal species communities, and mainly inhabit crenal sections of cold streams. Larval *Drusus crenophylax* were collected at eucrenal sections of the Cvrcka River (Fig. 6A, B) and behave as epilithic grazers, as indicated by mandible morphology (Pauls et al. 2008, Graf et al. 2009). Based on regional collection data, we assume that the species is a micro-endemic restricted to the watershed of the Cvrcka river.

#### Etymology.

The species epithet is a compound name, combining κρηνον (‘well, spring, fountain’ in Ancient Greek) and φυλαξ (‘guard, keeper, protector’ in Ancient Greek), terms that reflect the high degree of niche specificity of *Drusus* species, the majority of which inhabit crenal sections of streams (Graf et al. 2008).

### Key to Drusinae larvae of the *bosnicus*-group

Drusinae have evolved into three distinct subclades reflecting feeding ecology of larvae (Pauls et al. 2008, Graf et al. 2009). The grazer clade *sensu* Pauls et al. 2008 represents the largest clade, comprising over 70 species in several subclades (Malicky 2004, 2005; Kučinić et al. 2011a; Oláh 2010, 2011; Oláh and Kovács 2013). Larvae of scraping grazers species characteristically develop toothless mandibles (Pauls et al. 2008, Graf et al. 2009, Waringer and Graf 2011). In the Western Balkans, the grazing *bosnicus*-group represents a group of morphologically similar endemics and comprises according to Marinković-Gospodnetić (1976) *Drusus bosnicus* Klapálek, *Drusus klapaleki* Marinković-Gospodnetić, *Drusus medianus* Marinković-Gospodnetić, *Drusus plicatus* Radovanović, *Drusus radovanovici* (Marinković-Gospodnetić), *Drusus ramae* Marinković-Gospodnetić, *Drusus septentrionis* (Marinković-Gospodnetić) and *Drusus vespertinus* Marinković-Gospodnetić (Kučinić et al. 2011a).

Larvae of the *bosnicus*-group also develop, with the exception of *Drusus ramae* (Kučinić et al. 2010), a field of microspinules close to each eye (Kučinić et al. 2011a, b; Waringer et al. 2015). Further, carinae of *Drusus bosnicus*, *Drusus radovanovici*, *Drusus septentrionis* and *Drusus medianus* are high and curved mediad. Larvae of *Drusus crenophylax* sp. n. share those characters and can be integrated in the following dichotomous key (Waringer et al. 2015):

**Table:** 

1	Head with flat vertex	***Drusus bosnicus*** (Kučinić et al. in press)
–	Vertex evenly rounded	**2**
2	Pronotum with thin long, yellow setation	***Drusus radovanovici*** (fig. 17 in Kučinić et al. 2011a)
–	Pronotum without thin long, yellow setation	**3**
3	Pronotum with numerous short, white, recumbent setae	**4**
–	Pronotum without numerous short, white, recumbent setae	***Drusus septentrionis*** (figs 4, 5 in Kučinić et al. 2008)
4	Dorsal pronotal hump smoothly rounded	***Drusus medianus*** (fig. 43 in Kučinić et al. 2010, figs 20–22 in Kučinić et al. 2011b)
–	Dorsal pronotal hump with distinct ridge	**5**
5	Anterior metanotal sclerites narrowly subtriangular (width / length ratio ≥ 2.0)	***Drusus vespertinus*** (Previšić et al. 2009a)
–	Anterior metanotal sclerites broadly subtriangular (width / length ratio < 2.0)	**6**
6	In lateral view, dorsal pronotal ridge annular, posterior section sharply descending	***Drusus serbicus*** (Waringer et al. 2015)
–	In lateral view, posterior section of dorsal pronotal ridge gently descending	7
7	White recumbent setae cover the entire pronotum	***Drusus klapaleki*** (Kučinić et al. 2011b)
–	White recumbent setae lacking in a semicircular area anterior to the pronotal ridge	***Drusus crenophylax* sp. n.**

## Discussion

### Systematic significance of *Wormaldia sarda* sp. n.

The Tyrrhenian islands and Sardinia in particular have been renowned for their relictual fauna and flora for a long time (Holdhaus 1924) and represent one of the Mediterranean biodiversity hotspots (Grill et al. 2007). *Wormaldia sarda* sp. n. represents an addition to the distinct Sardinian biodiversity. As no species similar to *Wormaldia sarda* sp. n. are recorded from neither northern Africa nor mainland Europe, it is likely that this species is restricted to Sardinia, as are several other species such as *Crunoecia irrorata sarda* Curtis, *Stactobia ericae* Malicky or *Hydropsyche sattleri* Tobias (Graf et al. 2008). However, the geological history and geographic proximity of the Tyrrhenian islands – Sardinia and Corsica in particular (Vigliotti et al. 1990) – suggest that some species may occur on both islands. For instance, *Leptodrusus budtzi* Ulmer or *Micrasema togatum* Hagen occur also on Corsica, or other Mediterranean islands (Graf et al. 2008).

The distinct apomorphic characters, particularly the modified segment X and the very different pupal characters (mandibles, dorsal abdominal sclerites; Fig. 2D–F), might warrant establishing a new genus for this species. The pupal characteristics alone are strikingly different from those of either *Wormaldia* or *Philopotamus* (Lepneva 1964; Fig. 2). However, since pupae of only three species of *Wormaldia* are described (Nielsen 1942, Lepneva 1964) the range of genus-level pupal characters remains unknown. Further, modifications of segment X are common in southeast Asian species of *Wormaldia* (Malicky 2010). Tooth-like structures on segment X similar to the ones observed in *Wormaldia sarda* sp. n. are present in *Wormaldia* species from Thailand (e.g., *Wormaldia acheloos* Malicky & Chantaramongkol, *Wormaldia congina* Malicky & Chantaramongkol, *Wormaldia lot* Malicky & Chantaramongkol), or Sulawesi (*Wormaldia otaros* Neboiss). Nevertheless, *Wormaldia* species with a phallus shaped as in *Wormaldia sarda* sp. n. have not yet been described. Since the whole genus is in need of revision (Malicky 2005, Malicky unpubl. data), we refrain, in the interest of taxonomic stability, from creating a new genus.

### Aquatic diversity of the Western Balkans under threat

Endemic freshwater species are particularly vulnerable to global change and (anthropogenic) habitat degradation (Hering et al. 2009, Tierno de Figueroa et al. 2010, Bálint et al. 2011, Conti et al. 2014). The Balkans is rich in apparently endemic freshwater species (Griffiths et al. 2004). Recent taxonomic efforts in the Western Balkans increased the number of endemic Drusinae taxa to 31 of 39 described Drusinae species (Previšić et al. 2014b, Vitecek et al. unpubl. data). Further, several endemic species of *Chaetopteryx* were recently described from the Western Balkans (Oláh et al. 2012, Kučinić et al. 2013) indicating the need for further systematic investigations on an underestimated diversity of southeastern Europe.

The construction of hydropower dams in emerging economies is currently one of the greatest threats to freshwater biodiversity (Zarfl et al. 2014). Small hydropower plants fed by small cold-water mountain rivers such as the Cvrcka River are currently under construction throughout the Western Balkans (Freyhof 2012, Schwarz 2012), and gravely threaten the habitats that harbour endemic highland caddisflies such as Drusinae (Previšić et al. 2014a, Vitecek et al. unpubl. data, this study), or *Chaetopteryx* species (Kučinić et al. 2013). The description of *Drusus crenophylax* sp. n. highlights the importance of biodiversity research in southern Europe, and demonstrates that the currently prevailing energy policy will likely result in the loss of known and unknown biodiversity.

## Supplementary Material

XML Treatment for Wormaldia sarda

XML Treatment for Drusus crenophylax
